# Vague neuroglycopenic complaints camouflage diagnosis of adolescent insulinoma: a case report

**DOI:** 10.1186/s13633-016-0032-8

**Published:** 2016-07-29

**Authors:** Kelsee Halpin, Ryan McDonough, Patria Alba, Jared Halpin, Vivekanand Singh, Yun Yan

**Affiliations:** 1 Division of Endocrinology and Diabetes, Children’s Mercy Hospital, 2401 Gillham Rd, Kansas City, MO 64108 USA; 2 Department of Radiology, Saint Luke’s Hospital, 4401 Wornall Rd, Kansas City, MO 64111 USA; 3 Pathology and Laboratory Medicine, Children’s Mercy Hospital, 2401 Gillham Rd, Kansas City, MO 64108 USA

**Keywords:** Adolescent, Child, Altered mental status, Hypoglycemia, Epilepsy, Hyperinsulinism, Insulinoma

## Abstract

**Background:**

Insulinoma is a rare diagnosis in the general population with estimates of 1 in 250,000 people per year. Reports of these pancreatic islet cell tumors are even more unusual in children and adolescents. Chronic hypoglycemia due to an insulinoma often presents with neuroglycopenic symptoms that can easily be overlooked, especially in adolescents where nonspecific complaints are common. This may result in delayed diagnosis with prolonged periods of untreated hypoglycemia and associated complications. The rarity of pediatric insulinoma, vagueness of presenting symptoms, and challenge of tumor localization make insulinoma a true diagnostic quandary for clinicians.

**Case Presentation:**

In this report, we present a 15-year-old female who visited her primary care provider complaining of intermittent episodes of altered mental status including fatigue, irritability, and poor concentration. Her outpatient management included routine laboratory studies, drug screening, electroencephalogram (EEG), valproic acid initiation, CT scan of the abdomen, and endoscopic ultrasound with documentation of hypoglycemia, but otherwise inconclusive results. The patient was admitted to a tertiary children’s hospital with severe refractory hypoglycemia 8 months after the initial evaluation. A serum critical sample was obtained and magnetic resonance imaging (MRI) of the abdomen performed which confirmed the presence of a pancreatic mass ultimately identified as an insulinoma. She went on to have surgical resection of her tumor resulting in complete resolution of her hypoglycemia and associated symptoms.

**Conclusion:**

Within this report we demonstrate the importance of being vigilant for fasting hypoglycemia secondary to insulinoma even when the patient presents with nonspecific symptoms such as fatigue, irritability, or problems with concentration. If these neuroglycopenic complaints are unnoticed or misdiagnosed, patients with a potentially curable disease are put at risk of neurologic injury, or even death, due to untreated severe hypoglycemia.

## Background

An adolescent presenting with changes in behavior is not unusual in pediatric practice. Common suspects are routine teenage behavior, mental illness, or substance abuse. On the contrary, hypoglycemia, especially due to an insulin producing tumor, can easily be overlooked. Despite being the most common functioning neoplasm of the pancreas, insulinomas are rare in the general population with estimates at 1 in 250,000 people per year [1]. Epidemiology studies in pediatrics are even more limited. Insulinomas are comprised of pancreatic beta islet cells that produce excess amounts of endogenous insulin [2]. This inappropriate insulin secretion results in hypoglycemia with its associated neurogenic and neuroglycopenic symptoms. With prolonged and repeated hypoglycemia, neurogenic symptoms are minimized and nonspecific neuroglycopenic symptoms predominate [3]. We present the case of an adolescent female who delayed seeking medical attention given the ambiguity of symptoms and once assessed by a medical provider had a prolonged evaluation resulting in curative surgery more than 8 months after symptom onset. This case highlights the importance of awareness and recognition of neuroglycopenic symptoms in order to prevent undetected chronic hypoglycemia.

## Case Presentation

**Fig. 1:**
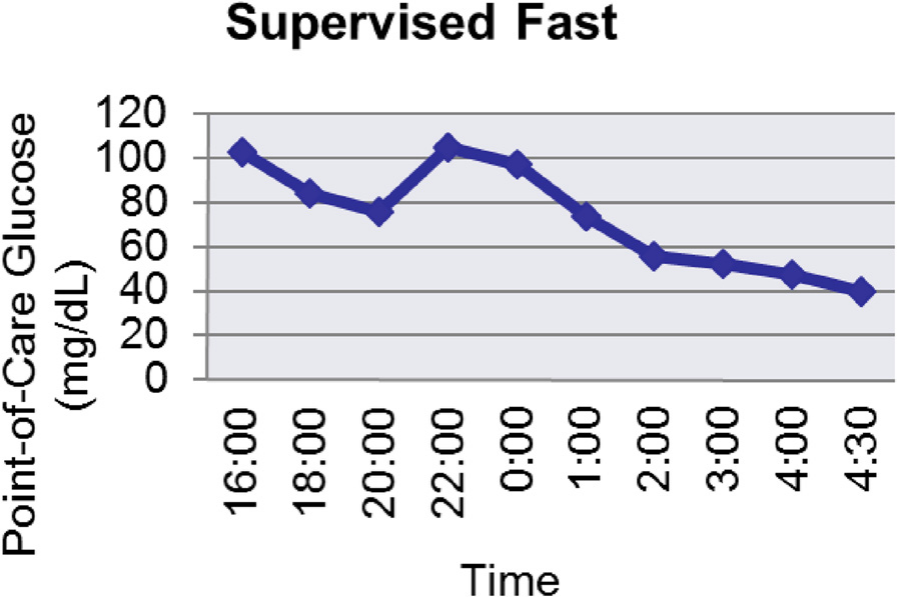
Point of care glucose trend during supervised fast. Our patient’s point-of-care glucose monitoring during hospital admission showing a rapid decline to 40 mg/dl 6 h after initiation of fasting at 22:00

**Fig. 2:**
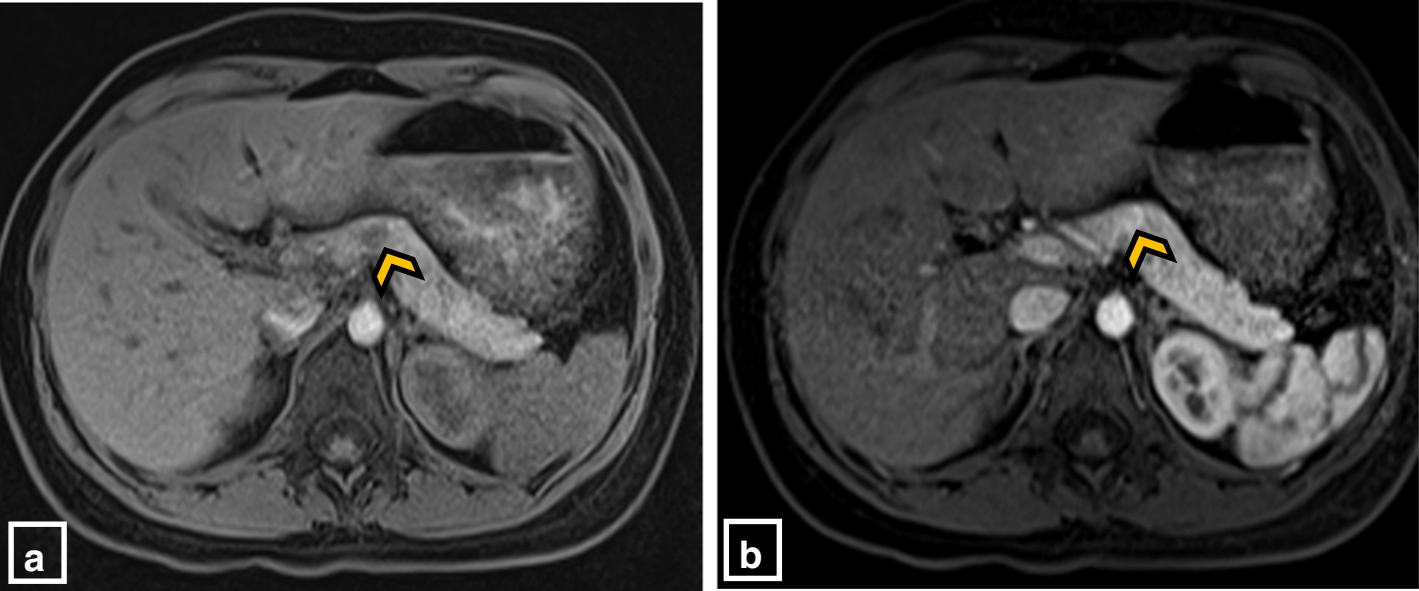
Result of MRI of the abdomen. Axial T1 weighted pre-contrast (**a**) and post-contrast (**b**) MRI images demonstrate a round enhancing lesion in the body of the pancreas (arrowhead)

**Fig. 3:**
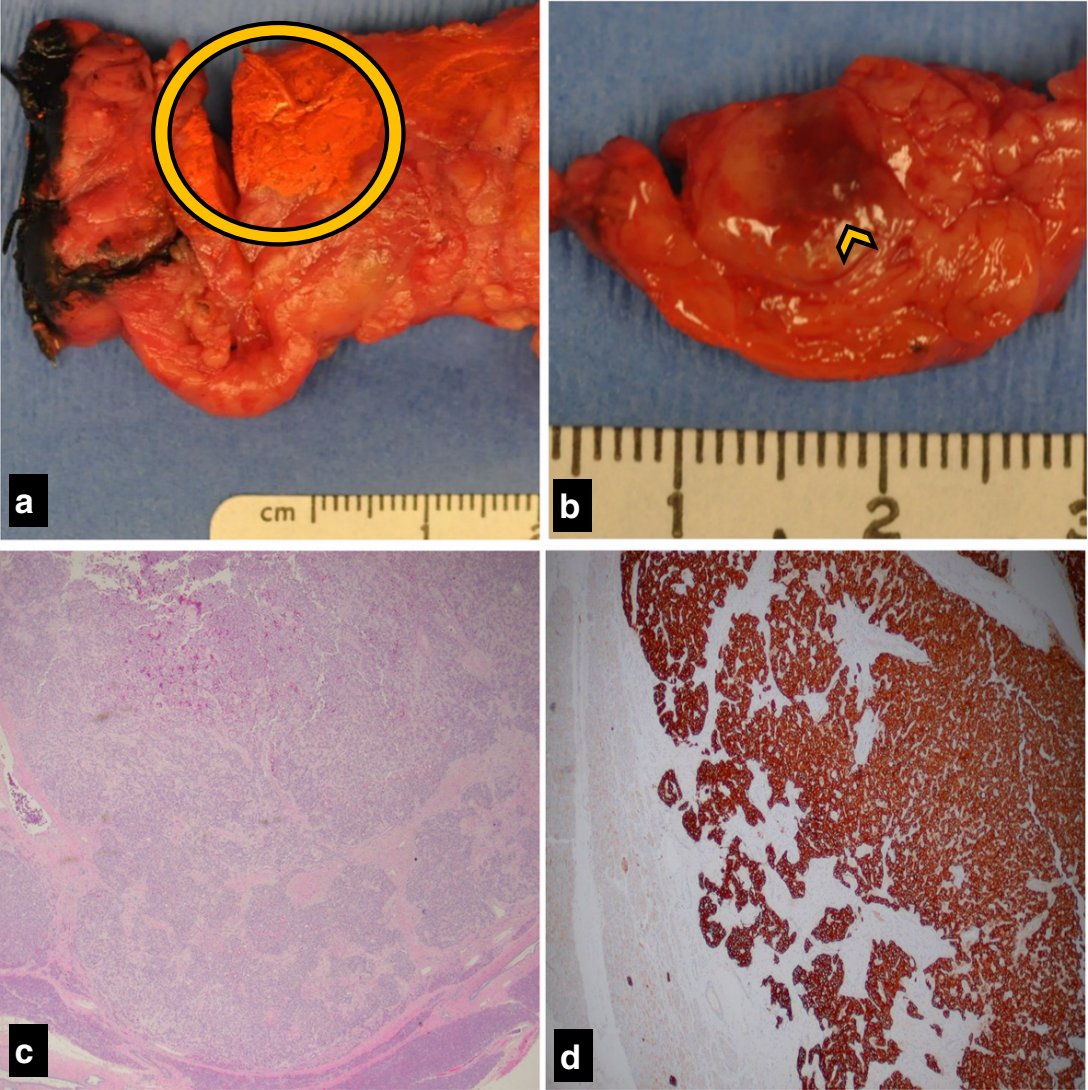
Gross and microscopic images of resected specimen. **a** Gross pancreatic specimen following laparoscopic distal pancreatectomy. External surface showing orange ink applied to the area in which a palpable mass was appreciated (circle). **b** Gross pancreatic body specimen showing cut surface with 1 cm hyperpigmented nodule (arrowhead), which presents a smooth surface distinct from adjacent normal lobulated pancreas. **c** Histologic section (H&E stain, 40x magnification) of the tumor showing an intact capsule separating it from normal pancreatic tissue. **d** Synaptophysin immunostain: diffuse positive staining present in tumor cells

A 15-year-old girl presented to her primary care provider with episodes of altered behavior. She reported a 30-minute period of fatigue, confusion, poor concentration, irritability, and “staring off” which was noted by parents while the patient was getting ready for school. The patient herself did not recall the event. Her parents had witnessed several similar events lasting up to 30 min, at least once per week, in the morning, for the past 5 months. Their daughter’s symptoms would resolve after eating breakfast. They had not sought medical care previously, assuming her behavior was related to early morning drowsiness. When the frequency of these episodes increased to several times per week, they presented to their primary care physician. Following her primary care visit, the patient was scheduled for an outpatient EEG after BMP, thyroid studies, and UDS which were normal. The patient became unresponsive during the EEG study and was referred to the local hospital with the following laboratory data when she was symptomatic: point-of-care glucose 36 mg/dL (65–110), insulin 36.8 uIU/mL (2.6–24.9), C-peptide 4.6 ng/mL (1.1–4.4), and betahydroxybutyrate 0.6 mg/dL (0.2–2.8). Her mental status immediately improved with intravenous dextrose. In search of an insulin-producing mass, she underwent abdominal CT with contrast which was negative per the local radiologist’s report. Her EEG was interpreted as abnormal prompting the initiation of valproic acid therapy for potential seizure activity. She was discharged home from the emergency room with a glucometer and planned neurology follow-up. An outpatient endoscopic ultrasound was pursued as another attempt to localize any potential insulin producing mass, and this too was unremarkable. She was subsequently referred for subspecialty consultation with a pediatric endocrinologist. Prior to her scheduled endocrinology appointment, she was admitted to a tertiary care pediatric center due to near daily episodes of hypoglycemia associated with altered mental status. The patient became hypoglycemic overnight while fasting in less than 6 h (Fig. 1). A critical sample was obtained and confirmed hyperinsulinemic hypoglycemia (Table 1). Testing for sulfonylurea ingestion was performed and the result was negative. An MRI of the abdomen revealed a 0.9 × 1.1 × 1.3 cm pancreatic mass (Fig. 2). Valproic acid was stopped and her hypoglycemia medically managed successfully with diazoxide 150 mg orally 3 times per day (8 mg/kg/day) and two tablespoons of cornstarch at bedtime. She ultimately underwent laparoscopic distal pancreatectomy with pathologic examination of the mass confirming a benign insulinoma (Fig. 3). She had mild hyperglycemia post-operatively which resolved within 48 h without insulin therapy and was discharged home on post-operative day three. Gene sequencing by using DNA from a peripheral blood sample for multiple endocrine neoplasia was negative.. The patient remained euglycemic, asymptomatic, and lost 15 kg in the 10 months following tumor resection which dropped her BMI from the 87th to 45th percentile. A follow up EEG was performed and was completely normal. She is now considered cured. The time from onset of symptoms to correct diagnosis, tumor localization, and surgical management was approximately 8 months.

**Table 1:** Laboratory evaluation.

Test (unit)	Result (reference range)
A.	
Glucose (mg/dL)	43 (65–110)
Insulin (mcIU/mL)	38.1 (2–18)
C-peptide (mcIU/mL)	4.1 (0.6–6.3)
Beta-Hydroxybutyrate (mcmol/L)	<100 (0–269)
Cortisol (mcg/dL)	4.4 (7–25)
Free fatty acids (mmol/L)	0.08
Human growth hormone (ng/mL)	0.2 (0–7)
B.	
Ammonia (mcmol/L)	<9 (4–33)
TSH (mcIU/mL)	2.19 (0.35–5.5)
Free T4 (ng/dL)	1.0 (0.8–1.9)
Cortisol 60 min after 250mcg cosyntropin (mcg/dL)	22.1
Sulfonylurea serum level	Undetectable
Insulin antibody level	Undetectable

A. Critical sample results reveal hyperinsulinemic hypoglycemia.

B. Additional studies obtained when euglycemic

## Discussion

Insulinomas are tumors composed of pancreatic beta-islet cells which produce excess endogenous insulin resulting in intermittent hypoglycemia. Epidemiologic studies in the United States estimate the incidence at approximately four insulinoma cases per million patients per year. The typical age at diagnosis is reported in the fifth decade of life with a slightly higher female prevalence [1]. However, insulinomas have been diagnosed in patients of all ages, including children and adolescents, though congenital hyperinsulinism remains the most common etiology for pediatric hyperinsulinemic hypoglycemia. Given the rarity of insulinoma in youth, its incidence in children has not been estimated [4]. Insulinomas are most often small (diameter less than 2 cm), localized, benign, and sporadic [5]. However, up to 10 % of insulinomas occur as a component of multiple endocrine neoplasia type 1 (MEN1), an autosomal dominant disorder resulting from a mutation in the MEN1 gene [1]. The list of MEN1 mutations discovered continues to grow, with over 1,300 mutations currently reported. [6].

Despite their typically benign anatomic characteristics, the functionality of insulinomas can result in significant morbidity. As a result of their unregulated insulin secretion, the patients with insulinoma have impaired hepatic glycogenolysis, gluconeogenesis, and ketogenesis resulting in severe, recurrent hypoglycemia. In response to the hypoglycemia two categories of symptoms are observed: neurogenic and neuroglycopenic (Table 2) [7]. Neurogenic symptoms often can be quickly be identified as clinicians and even patients associate them with low blood sugar. However, with prolonged, chronic hypoglycemia the adrenergic response can be blunted, resulting in a phenomenon known as “hypoglycemia-associated autonomic failure.” This occurs primarily because the glycemic threshold for initiation of the autonomic response is shifted to a lower serum glucose concentration. This results in patients having hypoglycemia unawareness with the first evidence of hypoglycemia being neuroglycopenic symptoms. Neuroglycopenic symptoms are a result of inadequate glucose for utilization by cerebral tissues and can be exceptionally vague. However, if not addressed, mild symptoms can progress to the most severe findings of neuroglycopenia, including coma and death [3].

**Table 2:** Symptoms of hypoglycemia: neurogenic vs. neuroglycopenic

Neurogenic Symptoms (Autonomic Nervous System Stimulation)	Neuroglycopenic Symptoms (Inadequate Glucose for Cerebral Use)
Palpitations	Fatigue
Diaphoresis	Personality change
Tremulousness	Difficulty with speech or concentration
Anxiety	Irritability
Paresthesias	Headaches
Hunger	Seizures
Light-headedness	Coma

Due to the ambiguity of presenting symptoms, the diagnosis of insulinoma is often elusive. It is not uncommon for insulinoma patients to be diagnosed with some form of neurologic disorder which resolves once their insulinoma is found and appropriately resected. Common alternative diagnoses include seizure disorders and cerebrovascular accidents or transient ischemic attacks. Diagnosis can be delayed for years with one study describing a mean time to diagnosis of 24 months with a range from 1 month to 30 years from symptom onset to surgical resection in patients ranging from 17 to 59 years of age [8]. The pediatric population represents an even more difficult population for making the diagnosis based on neuroglycopenic symptoms. Younger children may not be able to appropriately describe their symptoms and therefore it is left to the caregivers and medical providers to interpret clinical signs [9]. For teenagers, changes in mood and behavior can often been be perceived as a routine part of adolescence. Even if their altered mental status is more pronounced, other diagnoses such as substance abuse, trauma, or epilepsy may be deemed more likely [10, 11].

Furthermore, if there is clinical suspicion for hyperinsulinemic hypoglycemia, laboratory confirmation itself can be difficult. The classic clinical diagnosis relies on satisfying the criteria of Whipple’s triad which include 1) low serum glucose 2) symptoms consistent with hypoglycemia, and 3) immediate resolution of symptoms following treatment with carbohydrate [12]. Laboratory assessment should include serum glucose, insulin, and C-peptide or pro-insulin levels which should demonstrate hypoglycemia with inappropriately normal or elevated insulin concentrations. Elevated C-peptide or pro-insulin levels support endogenous insulin production where as these values would be suppressed with exogenous insulin administration (factitious hypoglycemia) [13]. Prolonged fasting may be necessary to induce and capture the biochemical evidence necessary to document hyperinsulinemic hypoglycemia [14]. If identified, hyperinsulinemic hypoglycemia outside of the neonatal period is suggestive of insulinoma, though recurrent sulfonylurea ingestion and stimulating insulin receptor mutation can present with comparable laboratory findings [15].

Localization of the tumor represents an additional challenge in the diagnosis of insulinoma. Given their small size, multiple imaging modalities may be required before the tumor is located [16]. Techniques that have been used are transabdominal and endoscopic ultrasound, CT, MRI, octreotide scan, calcium stimulation with angiography, and more recently 18 F-DOPA PET/CT. No single modality has been proven the gold standard and influences such as cost, sensitivity, operator skill, and availability of equipment must be considered. Typically, noninvasive studies are pursued initially with some combination of ultrasound and CT or MRI. If these are unsuccessful then more invasive techniques may follow [4, 17–19]. Tissue confirmation of the diagnosis of insulinoma can be achieved by a needle-core biopsy, laparoscopic biopsy or surgical excision of the tumor. Histologically, insulinoma is comprised of monomorphic tumor cells with the nuclei demonstrating stippled “salt and pepper” chromatin pattern typical of neuroendocrine tumors. Expression of chromogranin A, synaptophysin, and insulin can be demonstrated by immunohistochemistry, which further aids in confirming the diagnosis [8, 11].

Surgical resection is the standard of care for definitive insulinoma management. However, initial treatment for childhood insulinoma may focus on dietary modification to prevent hypoglycemia [16]. Medical therapies such as diazoxide and octreotide have been used successfully to prevent hypoglycemia in the perioperative period and for patients who are poor surgical candidates [20, 21]. Pancreas sparing techniques, including tumor enucleation and partial pancreatectomies, performed either openly or laparoscopically have been described in children with overall excellent cure rates and low risk of morbidity and mortality [22–24].

## Conclusion

Despite its rarity, clinicians must consider insulinoma in the differential diagnosis of hypoglycemia when addressing nonspecific concerns such has fatigue, irritability, or problems with concentration. This is especially true if symptoms are exacerbated by periods of fasting, such as early in the morning or accompanied by excessive weight gain. Our case highlights that a high index of suspicion is particularly critical in the adolescent population where vague somatic complaints or changes in behavior can easily be disregarded by families and the clinician alike as inconsequential. It is absolutely paramount that providers be aware of not only the more recognizable neurogenic symptoms, but also the neuroglycopenic findings in hypoglycemia and educate their patients and families accordingly. Our patient also taught us that there must be consideration of hypoglycemia in all patients presenting with changes in mental status. The key to timely diagnosis of insulinoma is to consider the possibility of hypoglycemia early in the patient’s evaluation, because even then, the challenge of obtaining diagnostic laboratory assays and imaging studies remains. Disregarding or misconceiving insulinoma patients’ neuroglycopenic complaints will delay their diagnosis further and can result in devastating neurologic consequences due to their recurrent, untreated severe hypoglycemia.

## Abbreviations

18 F-DOPA PET/CT, fluorine F 18 fluorodopa positron emission tomography/computed tomography; BMI, body mass index; BMP, basic metabolic profile; CT, computed tomography; EEG, electroencephalogram; MEN1, multiple endocrine neoplasia type 1; MRI, magnetic resonance imaging; UDS, urine drug screen
